# The *FHY3*/*FAR1* Gene Family in Plants: Transposase-Derived Transcription Factors as Master Integrators of Light Signaling and Plant Development

**DOI:** 10.3390/plants15121776

**Published:** 2026-06-09

**Authors:** Hao Li, Lan Wei, Conghao Hong, Qingqing Huang, Zhimin Huang, Hongbo Gao

**Affiliations:** 1 National Engineering Research Center of Tree Breeding and Ecological Restoration, State Key Laboratory of Efficient Production of Forest Resources, College of Biological Sciences and Technology, Beijing Forestry University, Beijing 100083, China; 2 Department of Molecular Biology and Genetics, Weill Institute for Cell and Molecular Biology, Cornell University, Ithaca, NY 14850, USA; 3 National Peony Gene Bank & Luoyang Peony Industry Development Center, Luoyang 471002, China

**Keywords:** *FHY3*/*FAR1* gene family, transposase-derived transcription factors, phytochrome A signaling, light signaling, plant development, stress responses

## Abstract

The FAR-RED IMPAIRED RESPONSE 1 (FAR1) and FAR-RED ELONGATED HYPOCOTYL 3 (FHY3) transcription factors, together with other members of the FAR1-RELATED SEQUENCE (FRS) and FRS-RELATED FACTOR (FRF) families, represent a striking example of transposable element domestication in plants. Derived from ancient *Mutator*-like element (MULE) transposases, these proteins have been repurposed as transcriptional regulators throughout the plant kingdom. FHY3 and FAR1 were first identified in *Arabidopsis thaliana* as positive regulators of phytochrome A (phyA) signaling. They participate in the coordination of light signaling with the circadian clock, chlorophyll biosynthesis, hormone pathways, stress responses, flowering time, shoot branching, leaf senescence, seed dormancy, and phosphate homeostasis. At the molecular level, FHY3 and FAR1 regulate gene expression mainly by binding to the conserved FHY3/FAR1-binding site, FBS, with the sequence CACGCGC, in the promoters of target genes. They also act through protein interactions with key signaling regulators, including HY5, PIFs, EIN3, TOC1, and SPL transcription factors. In this review, we summarize the molecular basis of *FHY3*/*FAR1* gene family function, discuss the roles and mutant phenotypes of characterized family members, and highlight recent advances from other plant species beyond Arabidopsis. Collectively, this gene family illustrates how domesticated transposase-derived proteins have evolved into key regulators of plant development and environmental adaptation.

## 1. Introduction

Transposable elements (TEs) are mobile DNA sequences that can move within genomes. Through domestication, host organisms co-opt TE-encoded proteins for regulatory functions [1]. Transposases encode DNA-binding domains and protein–protein interaction interfaces that can be repurposed as transcription factors once catalytic activity is lost [2]. *Mutator*-like element (MULE)-derived *MUSTANG* genes regulate plant development [3], and Transib-derived recombination-activating gene 1 *(RAG1*) mediates variable (V), diversity (D), and joining (J) (V(D)J) recombination in animals [4]. The *FAR-RED IMPAIRED RESPONSE 1* (*FAR1*)/*FAR-RED ELONGATED HYPOCOTYL 3* (*FHY3*) family represents another well-characterized case of TE domestication.

Originally identified in screens for *Arabidopsis* phytochrome A signaling defects, FAR1 and FHY3 illustrate how a domesticated transposase was recruited into plant light signaling networks. Plants use photoreceptors to detect red and far-red light [5]. Among the five known *Arabidopsis thaliana* phytochromes (phyA–phyE), phytochrome A (phyA) uniquely mediates responses to the very low fluence response (VLFR) and the far-red high-irradiance response (FR-HIR), the latter of which requires continuous far-red light for sustained activation [6,7,8]. Forward genetic screens in *A. thaliana* designed to identify mutants impaired in phyA signaling led to the discovery of two genes encoding previously unknown nuclear proteins, *FAR1* and *FHY3* [5,9]. Notably, protein sequence analysis revealed that FAR1 and FHY3 share significant homology with MULE transposases of the *MuDR* family, establishing them as the best-characterized examples of transposable element (TE) domestication in eukaryotes [9,10].

Since their initial identification, the functional repertoire of FHY3 and FAR1 has expanded far beyond phyA signaling. As transcription factors, they not only directly regulate the expression of *FHY1* and *FHL*, which encode proteins essential for phyA nuclear import [10,11], but also govern many biological processes, including chlorophyll biosynthesis [12], the circadian clock [13], abscisic acid (ABA) signaling [14], shoot branching [15], flowering time [16,17], leaf senescence [18], seed dormancy [19], starch metabolism [20], UV-B responses [21], phosphate homeostasis [22], reactive oxygen species (ROS) scavenging [23] and plant immunity [24].

Beyond *FHY3* and *FAR1*, the family includes twelve *FAR1-RELATED SEQUENCE* (*FRS1*–*FRS12*) genes and at least four *FRS-RELATED FACTOR* (*FRF1*–*FRF4*) genes in *Arabidopsis* [25,26]. Although the functions of most FRS/FRF members remain unknown, recent studies have revealed distinct roles for specific members [17,26,27]. This gene family is widely conserved among angiosperms from a genome-wide analysis in crop species including soybean, maize, rice, quinoa, ginseng and eucalyptus, suggesting that its expansion is frequently driven by polyploidy [28,29,30,31,32].

In this review, we summarize how the *FHY3*/*FAR1* gene family has evolved into system integrators that connect light perception to hormone signaling, developmental timing, and stress adaptation. We discuss the evolutionary origin and protein architecture of these transposase-derived transcription factors, systematically review the functions and mutant phenotypes associated with each characterized family member, and survey the rapidly growing body of work in plants.

## 2. Evolutionary Origin and Domain Architecture of the *FHY3/FAR1* Family

### 2.1. Transposase Domestication and Conserved Protein Architecture

**Figure 1:**
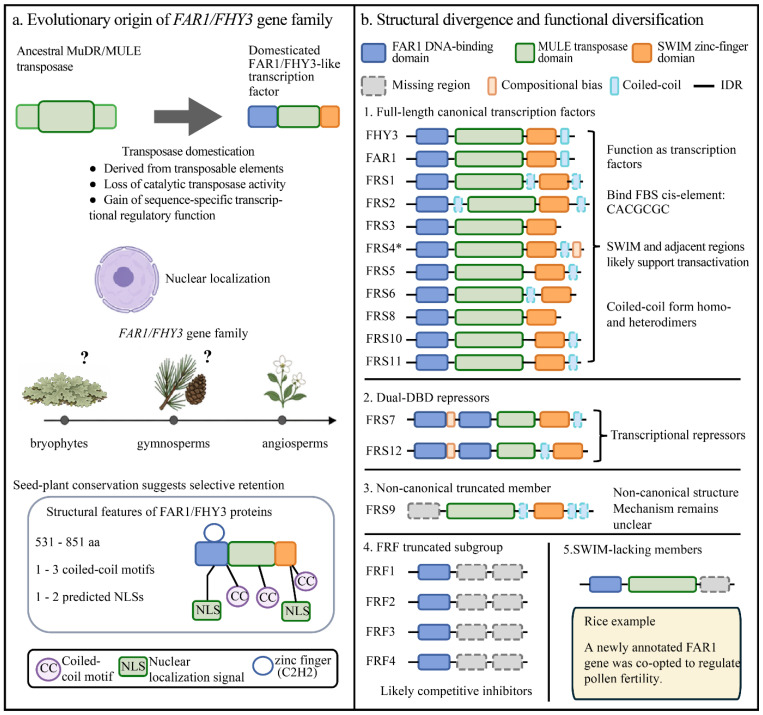
The evolutionary origin and protein structural diversification of the *FHY3*/*FAR1* family genes. (**a**) The *FHY3*/*FAR1* family genes originated from ancestral *MULE* transposases through domestication, during which transposase activity was lost and transcriptional regulatory function was acquired. Question marks indicate that the evolutionary relationships of the reported bryophyte and gymnosperm *FHY3*/*FAR1*-related sequences to angiosperm FHY3/FAR1 proteins require further clarification [33]. These angiosperm proteins encode nuclear-localized regulators with conserved DNA-binding, MULE-derived, SWIM, coiled-coil and NLS features. (**b**) The divergent architectures of FHY3/FAR1 family protein and regulatory functions in *Arabidopsis* and rice. Section 1, Section 2, Section 3 and Section 4 correspond to *Arabidopsis* members, whereas Section 5 represents a rice member. Full-length members mainly act as transcriptional activators, FRS7 and FRS12 function as repressors, whereas truncated FRF and SWIM-lacking proteins likely modulate or compete with canonical FHY3/FAR1 activity. The asterisk indicates that FRS4 acts as a passive or competitive transcriptional repressor in *Arabidopsis*. The domain architectures and structural features are schematic representations based on UniProt database annotations for representative family members and not drawn to scale.

FHY3 and FAR1 belong to a class of plant regulatory proteins derived from transposable elements (Figure 1a) [2,10]. Phylogenetic analyses place FAR1 and FHY3 within the *Mutator*-like element (MULE) superfamily, specifically the *MuDR* clade that also includes the maize *Mutator* transposon system (Figure 1a) [9,26]. In contrast to canonical transposases, however, FAR1 and FHY3 have lost catalytic activity and instead evolved as sequence-specific transcription factors (Figure 1a) [9]. FHY3/FAR1 proteins were documented in angiosperms, and FHY3/FAR1-related sequences were also reported in some bryophyte and gymnosperm genomes (Figure 1a) [10,33]. These observations suggest that this transposase-derived lineage may have an ancient origin. However, the evolutionary relationships between the reported bryophyte and gymnosperm sequences and angiosperm FHY3/FAR1 proteins remain insufficiently resolved and require further phylogenetic analysis and functional characterization. The conservation of well-characterized FHY3/FAR1 proteins in angiosperms suggests that, once recruited for regulatory functions, these transposase-derived proteins were subject to functional constraint (Figure 1a) [9,25]. Proteins in the FHY3/FAR1 family range from approximately 531 to 851 amino acids, and most members contain one to three coiled-coil motifs together with one or two predicted nuclear localization signals, consistent with the nuclear localization observed for proteins examined experimentally (Figure 1a) [25].

Full-length FHY3/FAR1 family members, including FHY3, FAR1, FRS1, FRS2, FRS3, FRS4, FRS5, FRS6, FRS8, FRS10 and FRS11, share a conserved tripartite domain organization (Figure 1b) [10,26]. The N-terminal region contains a C2H2 zinc-finger DNA-binding domain, termed the FAR1 domain, that recognizes the FHY3/FAR1-binding site (FBS; CACGCGC) in target promoters (Figure 1b) [34]. The central region corresponds to a putative MULE transposase-derived domain that contributes to protein–protein interaction and dimer formation, consistent with the ability of FHY3 and FAR1 to form both homodimers and heterodimers (Figure 1b) [5,9]. The C-terminal region harbors a SWIM zinc-finger domain with transcriptional activation activity (Figure 1b) [34]. Together, these features illustrate how an ancestral transposase scaffold was remodeled into a transcriptional regulator, although the precise biochemical mechanism by which the SWIM-containing C-terminal region promotes the assembly of the transcriptional machinery remains unresolved (Figure 1b).

### 2.2. Family Classification and Phylogeny

Phylogenetic analyses of the *Arabidopsis* FHY3/FAR1 protein family generally divide its members into six subgroups [25,26]. Subgroup I includes the major transcription factors: FHY3, FAR1, FRS1, FRS2, and FRS4 (Figure 1b) [26]. Subgroup II comprises FRS6 and FRS8, which are predicted to function as transcription factors, but remain less well-defined in terms of their specific targets (Figure 1b) [25]. Subgroup III is the best characterized clade including FRS7 and FRS12 (Figure 1b) [26]. Subgroup IV includes FRS3, FRS5, and FRS9, whose members are closely associated with the jasmonate signaling pathway [25]. Subgroup V consists of FRS10 and FRS11 with largely unknown biological functions, whereas Subgroup VI corresponds to the FRF proteins (Figure 1b) [25,26].

### 2.3. Domain Truncation and Functional Diversification

Truncated members of the FHY3/FAR1 protein family exhibit clear structural and functional divergence from the full-length transcriptional activators [10,26,27]. Compared with the FAR1 and FHY3, FRS7 and FRS12 have an additional FAR1 domain (Figure 1b) [26], and act as transcriptional repressors rather than activators [17]. FRS9 is even more divergent, lacking the entire FAR1 domain (Figure 1b) [26], implying the existence of a non-canonical mechanism that remains to be clarified (Figure 1b) [35]. The FRS-RELATED FACTOR proteins, FRF1-FRF4, are further truncated, lacking the MULE and SWIM domains, which were proposed to attenuate FHY3/FAR1 activity by competitively binding at FBS-containing promoters without activating transcription (Figure 1b) [26].

Beyond these *Arabidopsis* examples, large-scale comparative genomics indicate that recurrent frameshift and nonsense mutations have contributed substantially to structural diversification across the broader FAR1-derived family (Figure 1b) [26,27,36,37]. In some cases, these truncated proteins are defined as FM-type, retaining the FAR1 DNA-binding domain and MULE-derived region but lacking the C-terminal SWIM domain and its associated transcriptional activation function (Figure 1b) [38]. In rice, a newly annotated FM-structured *FAR1* gene generated by a species-specific frameshift mutation has been shown to regulate pollen fertility (Figure 1b) [36]. This provides direct evidence that *FAR1*-gene-truncated derivatives can acquire biologically important functions [36]. These findings indicate that the *FHY3*/*FAR1* gene family has undergone extensive protein structural remodeling during evolution [25].

## 3. Biological Functions of the *FHY3/FAR1* Gene Family in *A. thaliana*

### 3.1. Phytochrome A Signaling

**Figure 2:**
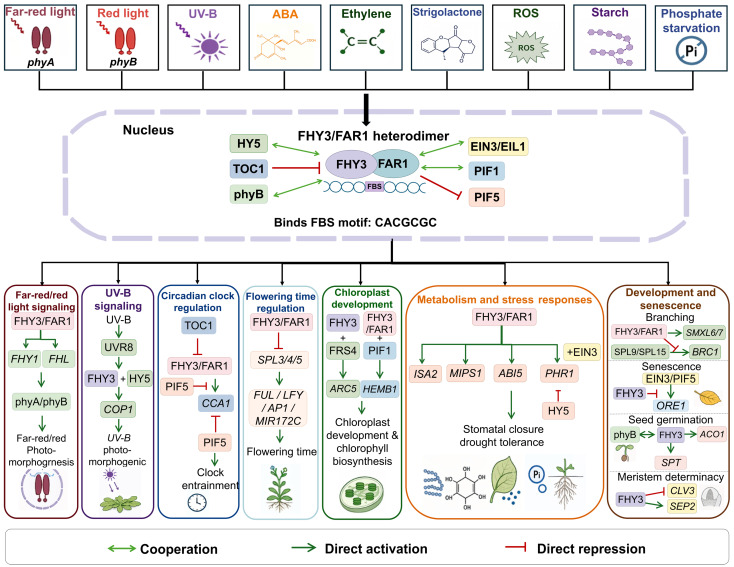
The FHY3/FAR1-mediated integration of light, hormone, metabolic and stress signals. FHY3 and FAR1 act as nuclear transcription factors that integrate multiple upstream signals, including far-red light, red light, UV-B, ethylene, ABA, strigolactone, phosphate starvation, sugar and ROS. In the nucleus, FHY3/FAR1 form heterodimers and bind to the FBS motif, CACGCGC, through the N-terminal DNA-binding domain. Their activity is modulated by interacting regulators such as HY5, TOC1, phyB, EIN3/EIL1, PIF1 and PIF5. Downstream, FHY3/FAR1 regulate diverse biological processes, including far-red/red light signaling, UV-B responses, circadian clock regulation, flowering time, chloroplast development, metabolism, stress responses, meristem determinacy, branching, senescence and seed germination. The green arrows indicate direct activation or cooperation, whereas the red lines indicate direct repression.

The function of FHY3/FAR1 is best-established in phytochrome A (phyA) signaling [5]. PhyA is synthesized in the cytoplasm as the inactive Pr form and converted to the active Pfr form upon far-red light activation, which imports into the nucleus via FHY1/FHL proteins through an importin-dependent pathway to directly regulate gene expression (Figure 2) [10,11,39]. FHY3 and FAR1 directly activate the transcription of *FHY1*/*FHL* by binding to FBSs in their promoters (Table 1), thereby promoting phyA nuclear accumulation and downstream far-red responses (Figure 2) [10]. Consistent with this role, the *fhy3* mutant exhibits severe defects in far-red responses: the *far1* mutant shows a weaker but related phenotype, and the *fhy3 far1* double mutant is more strongly impaired than either single mutant, indicating partial functional redundancy between the two factors [5,9,10]. These relationships are consistent with the original mechanistic studies on FHY3/FAR1, the role of FHY1/FHL in phyA nuclear accumulation, and later work linking FHY3/FAR1 to interacting regulators in related light signaling contexts [5,11,40,41].

This transcriptional module is further refined by feedback and post-translational control. ELONGATED HYPOCOTYL5 (HY5) physically interacts with FHY3/FAR1 and attenuates their activation of *FHY1*/*FHL*, thereby forming a negative-feedback loop that helps maintain phyA signaling homeostasis [42]. In addition, PHYTOCHROME INTERACTING FACTOR (PIF) family members can interact with FHY3 and modulate its transcriptional activity at shared target promoters [12,43]. Far-red signaling is also tuned at the post-translational level through reversible SUMOylation of FHY1 [44]. Far-red light promotes SUMOylation at K32 and K103 accelerating FHY1 degradation, whereas the SUMO protease ASP1 counteracts this process through deSUMOylation and stabilization of FHY1 [44]. Together, these findings show that FHY3 and FAR1 regulate phyA signaling through both transcriptional activation of the nuclear import machinery and integration of a broader regulatory mechanism, which fine-tune pathway output.

### 3.2. UV-B Signaling and COP1 Regulation

FHY3 also contributes to ultraviolet B (UV-B) signaling, extending its regulatory function beyond visible light responses [21,45]. In this pathway, UV-B is perceived by the photoreceptor UVR8 and triggers photomorphogenic responses mediated through COP1 and HY5 (Figure 2). FHY3 activates *COP1* transcription via the FBS element in response to UV-B (Figure 2, Table 1) [21,25]. This activation occurs cooperatively with HY5 and requires a functional UVR8 pathway, indicating that FHY3 acts as part of a broader transcriptional module controlling UV-B-induced photomorphogenesis (Figure 2) [21]. Consistent with this role, the *fhy3* mutant shows a reduction of UV-B-induced gene expression together with impaired photomorphogenic responses under UV-B conditions [21]. These findings place FHY3 upstream of both COP1 and HY5 in this signaling context and further support the view that FHY3 functions as a versatile integrator of distinct light signaling pathways rather than as a regulator restricted to far-red light responses [21,46].

### 3.3. Regulation of the Circadian Clock

FHY3 and FAR1 also play an important role in circadian clock regulation by linking light signaling to the transcriptional control of core oscillator genes (Figure 2) [13,43,47]. A key advance in this area was the demonstration that FHY3 and FAR1 directly bind the promoter of *CIRCADIAN CLOCK ASSOCIATED 1* (*CCA1*) and activate light-induced *CCA1* expression (Table 1), thereby establishing a direct molecular link between phytochrome signaling and the *Arabidopsis* circadian clock (Figure 2) [43]. This activation is not constitutive but is instead controlled by antagonistic regulators that determine the temporal pattern of *CCA1* transcription. Firstly, PIF5 directly binds to the *CCA1* promoter and represses its expression, while also physically interacting with FHY3 to inhibit its transcriptional activation activity (Figure 2) [43]. Furthermore, TIMING OF CAB EXPRESSION 1 (TOC1) exerts a similar antagonistic effect by interacting with FHY3 and attenuating its activity at the *CCA1* promoter (Figure 2) [43,48,49]. Phosphorylation of TOC1 enhances its affinity for FHY3, indicating that post-translational regulation further adjusts the strength of this repressive interaction across the diurnal cycle [49]. Thus, FHY3/FAR1-mediated activation and PIF5/TOC1-mediated repression together form an antagonistic regulatory module that integrates light input with endogenous circadian timing and helps generate robust rhythmic expression of *CCA1* [13,43].

### 3.4. Regulation of Flowering Time

FHY3 and FAR1 regulate flowering time by integrating light cues with the age-dependent miR156-SPL pathway (Figure 2) [15,16,25,50,51]. Mechanistically, FHY3 and FAR1 physically interact with the flowering promoting transcription factors SPL3, SPL4 and SPL5, and inhibit their association with the promoters of key floral transition genes, including *FRUITFULL (FUL)*, *LEAFY (LFY)*, *APETALA1 (AP1)* and *MIR172C* (Figure 2) [16,51]. Through this inhibitory action, FHY3 and FAR1 suppress the transcriptional output of SPL-mediated floral induction and thereby delay the transition from vegetative to reproductive development [16]. This regulatory module is particularly important under shade conditions, where FHY3 and FAR1 protein abundance declines whereas SPL3, SPL4 and SPL5 accumulate, resulting in increased expression of downstream floral promoting genes and accelerated flowering [16]. Thus, FHY3 and FAR1 provide a mechanistic link between light signaling, the aging pathway and shade-avoidance-associated reproductive timing, helping plants coordinate flowering with changes in the canopy light environment [16,52].

### 3.5. Chlorophyll Biosynthesis and Chloroplast Development

Beyond phyA signaling, FHY3 and FAR1 also function directly in chlorophyll biosynthesis and chloroplast development (Figure 2). FHY3 binds to the promoter of *HEMB1*, which encodes 5-aminolevulinic acid dehydratase, a key enzyme in tetrapyrrole biosynthesis, and cooperates with PIF1 to regulate its expression during de-etiolation (Figure 2, Table 1) [12]. Consistent with this role, *fhy3* mutants show reduced *HEMB1* expression and impaired chlorophyll accumulation during the dark-to-light transition [12]. FHY3 also promotes chloroplast division by activating *ARC5*, which encodes a dynamin-related protein required for chloroplast division, and this regulation is further supported by FRS4 through binding to FBS and FBS-like elements in the *ARC5* promoter (Figure 2, Table 1) [53,54,55]. Importantly, this branch of regulation is genetically separable from phyA signaling, because *arc5* mutants display normal far-red responses and the overexpression of *FHY1* does not rescue the chloroplast division defect [53].

The function of FHY3/FAR1 in chlorophyll metabolism is also closely linked to redox homeostasis and immunity. The *fhy3 far1* double mutant accumulates elevated reactive oxygen species and salicylic acid, accompanied by constitutive defense gene expression and enhanced resistance to *Pseudomonas syringae* [23,24]. This phenotype has been linked to chloroplast-derived retrograde signaling, supporting a role for FHY3/FAR1 as negative regulators of salicylic acid-mediated defense [24]. In parallel, FHY3 and FAR1 directly activate *MIPS1*, thereby promoting myo-inositol biosynthesis and contributing to oxidative stress protection (Figure 2, Table 1) [23]. Together, these findings indicate that FHY3 and FAR1 coordinate chloroplast biogenesis with metabolic and defense responses [12,23,24,53,54].

### 3.6. ABA Signaling and Abiotic Stress Responses

FHY3 and FAR1 also function at an important point of convergence between light signaling and abscisic acid (ABA) responses (Figure 2) [14,46,56,57]. They directly bind to the promoter of *ABA INSENSITIVE 5 (ABI5)* (Table 1), a central transcriptional regulator in the ABA signaling pathway, and activate its expression (Figure 2) [14]. Consistent with this transcriptional role, the loss of FHY3 or FAR1 compromises ABA responsiveness, and the *fhy3* and *far1* mutants display reduced sensitivity to ABA-induced stomatal closure, increased stomatal aperture, faster transpirational water loss and greater drought sensitivity than wild-type plants [14]. In addition, expression analyses have shown that FHY3 and FAR1 themselves respond to ABA and abiotic stress conditions, further supporting their participation in stress-associated signaling networks (Figure 2) [14,56]. Together, these findings indicate that FHY3 and FAR1 act as signal integrators to positively regulate ABA-mediated drought responses and illustrate how light-responsive transcription factors can be recruited to coordinate growth control with environmental stress adaptation [14,58].

### 3.7. Strigolactone Signaling

Shoot branching is a major determinant of plant architecture and productivity, and FHY3 and FAR1 have emerged as key integrators of light quality and strigolactone (SL) signaling in the control of this process (Figure 2) [15]. In the SL pathway, the TCP transcription factor BRC1 acts as a central suppressor of branch outgrowth in axillary buds [59]. FHY3 and FAR1 modulate this pathway through two coordinated mechanisms (Figure 2) [15,60]. On the one hand, they directly interact with SPL9 and SPL15 and inhibit their transcriptional activation of *BRC1*, thereby alleviating the repression of branching (Figure 2, Table 1) [15,59]. On the other hand, they directly promote the expression of *SMXL6* and *SMXL7*, which encode major repressors of SL signaling and further favor bud outgrowth (Figure 2, Table 1) [15,60]. Under simulated shade conditions, where the red to far-red ratio is reduced, FHY3 protein abundance declines, leading to enhanced *BRC1* activation together with reduced *SMXL6*/*7* expression, and consequently to stronger SL-mediated inhibition of branching [15]. This regulatory module provides a molecular explanation for the reduced lateral branching observed during shade avoidance.

### 3.8. Leaf Senescence

Leaf senescence is controlled by the coordinated action of developmental age, environmental signals, and phytohormone pathways, and FHY3 and FAR1 function as important negative regulators of its onset (Figure 2) [18,46,61]. Genetic and molecular studies have shown that these factors participate in an age-gating mechanism that prevents premature senescence in young leaves [18,46]. In developing green leaves, FHY3 accumulates to levels sufficient to antagonize the activities of EIN3 and PIF5 at the *ORE1* promoter, thereby repressing the expression of this central positive regulator of senescence (Figure 2, Table 1) [46,61]. As leaves age or are exposed to prolonged darkness, the abundance of FHY3 and FAR1 progressively decreases, which relieves this repression and allows EIN3 and PIF5 to promote *ORE1* expression and senescence progression [46]. In addition to this pathway, FHY3 directly represses *WRKY28* (Table 1), thereby limiting the *WRKY28*-dependent activation of *SID2*/*ICS1* and the associated salicylic acid biosynthetic pathway [18,46]. Together, these findings indicate that FHY3 and FAR1 integrate light and age-related information to delay senescence until leaves reach an appropriate developmental stage.

### 3.9. Seed Dormancy and Germination

FHY3 also plays a central role in the regulation of seed dormancy and germination by linking light perception to hormonal control pathways (Figure 2) [14,19,62,63]. It has been shown that FHY3 physically interacts with phyB both in vivo and in vitro and directly regulates the expression of *RVE2*, *RVE7,* and *SPATULA (SPT)* through binding to FHY3/FAR1-binding sites in their promoters (Figure 2, Table 1) [19]. These downstream regulators in turn affect the expression of *GA3ox2*, a key gibberellin biosynthetic gene, establishing a phyB-FHY3 RVE2/RVE7/SPT-GA3ox2 regulatory cascade that promotes light-induced germination [19,63]. More recent work further demonstrated that FHY3 contributes to dormancy release through modulation of ethylene biosynthesis [62]. Specifically, FHY3 directly activates *ACO1*, which encodes ACC OXIDASE 1 and catalyzes the conversion of ACC to ethylene, thereby linking light signaling with ethylene production in imbibed seeds (Figure 2, Table 1) [62]. These findings show that FHY3 promotes germination through coordinated regulation of both gibberellin- and ethylene-related pathways and highlight its broader function as an integrator of environmental light cues with endogenous hormonal signals.

### 3.10. Starch Metabolism and Phosphate Homeostasis

Starch biosynthesis and degradation are tightly coordinated with the light–dark cycle, and FHY3 and FAR1 contribute to this regulation by linking light signaling with carbohydrate metabolism (Figure 2) [20]. FHY3 and FAR1 positively regulate *ISOAMYLASE 2 (ISA2)*, which encodes components of the isoamylase-type debranching enzyme complex required for proper starch granule synthesis and turnover (Figure 2, Table 1) [20]. Accordingly, the *fhy3 far1* double mutant displays reduced starch accumulation together with altered granule morphology [20]. This indicates that these transcription factors are important for normal starch metabolism [20]. This regulatory process is further influenced by HXK1-dependent sugar sensing, providing a mechanism through which light and sugar status are integrated to fine-tune carbon storage [20,64]. In addition to their role in starch metabolism, FHY3 and FAR1 also participate in phosphate homeostasis by directly regulating PHOSPHATE STARVATION RESPONSE 1 (PHR1), a central transcriptional regulator of phosphate starvation responses (Figure 2, Table 1) [22]. At the PHR1 promoter, FHY3, FAR1, HY5, and EIN3 form a multilayered regulatory module in which FHY3, FAR1, and EIN3 function as activators whereas HY5 acts as a repressor [22]. Through this mechanism, plants are able to coordinate phosphate acquisition with light conditions and nutrient availability [20,22,65,66].

### 3.11. Floral Meristem Determinacy

In addition to its roles in environmental signaling, FHY3 also contributes to floral meristem determinacy through coordinated regulation of the genes controlling stem cell maintenance and floral organ specification (Figure 2) [67,68,69]. FHY3 directly represses *CLAVATA3* (*CLV3*), which encodes a signaling peptide that restricts stem cell proliferation, while at the same time activating *SEPALLATA 2* (*SEP2*), a MADS-box gene required for floral organ identity and meristem determinacy (Figure 2, Table 1) [67]. Through this dual regulatory activity, FHY3 promotes the transition from indeterminate meristem activity to determinate floral development [67,70]. Genetic analyses indicate that FAR1 plays a less prominent role than FHY3 in this process [12,67,71]. That suggests a degree of functional divergence between these two closely related paralogs beyond their shared functions in light signaling [46,57]. This work expands the functional scope of FHY3 from photomorphogenic regulation to meristem control and highlights how members of the *FHY3*/*FAR1* gene family can be recruited into core developmental programs [67,71].

Across the pathways described above, several common patterns of signaling crosstalk appear. FHY3 and FAR1 proteins often connect light input with hormone output by linking phytochrome signaling to ABA, ethylene, strigolactone, and jasmonate pathways. The same FHY3 or FAR1 protein can activate or repress different targets depending on the coregulator involved. For example, FHY3 activates *PHR1* together with EIN3, but it represses *ORE1* by counteracting EIN3 and PIF5. Developmental stage and light quality also serve as gating signals that determine when and where FHY3 and FAR1 interact with specific partners. These recurring patterns show that signaling crosstalk is not incidental. It is a feature of the interaction-based regulatory architecture of this protein family.

## 4. Functional Diversification of Other *A. thaliana* FRS and FRF Family Members

### 4.1. Subgroup I Members: FRS1, FRS2 and FRS4

**Figure 3:**
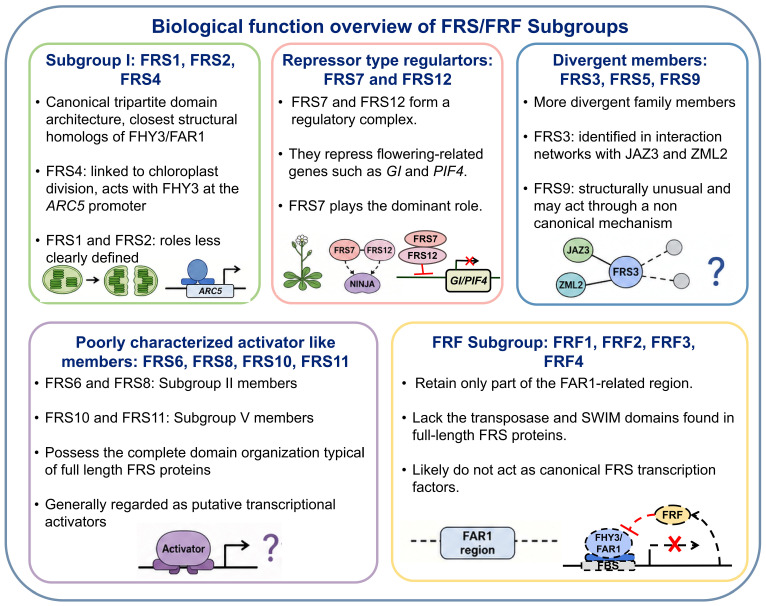
The biological functional diversification of *A. thaliana* FRS and FRF protein subfamily members other than FHY3 and FAR1. A summary of representative FRS and FRF proteins beyond FHY3 and FAR1 in *A. thaliana*, highlighting major functional categories, including Subgroup I members, the FRS7/FRS12 repressor complex, divergent signaling-related members, poorly characterized activator-like proteins, and the truncated FRF Subgroup. The solid lines indicate experimentally supported evidence, whereas the dashed lines indicate putative or inferred relationships. The arrows denote activation, and the blunt-ended arrows denote repression. The “X” denotes inhibition, and the “?”denotes unknown regulatory functions.

FRS1, FRS2 and FRS4 belong to Subgroup I and share the canonical tripartite domain architecture found in FHY3 and FAR1, suggesting that they represent the closest structural homologs of the two family members (Figure 3) [25,26]. Among them, FRS4 is the best characterized functionally. It has been linked to chloroplast division through cooperative action with FHY3 at the *ARC5* promoter, where the two proteins contribute to transcriptional regulation of a key chloroplast division gene (Figure 3) [54,72]. By contrast, *FRS1* and *FRS2* remain less functionally characterized, but their expression overlaps with *FHY3* and *FAR1* suggest potential redundant or context-dependent roles in light-regulated development (Figure 3) [25,26]. Direct downstream targets unique to FRS1 or FRS2 have not yet been identified, leaving it open whether they act redundantly with FHY3/FAR1 or have acquired context-specific target specificity [25]. Together, these observations suggest that Subgroup I contains both functionally established and still incompletely resolved members, with FRS4 providing the clearest evidence of diversification within this activator-like branch.

### 4.2. Repressor-Type Regulators: FRS7 and FRS12

In contrast to the activator-type members of the family, FRS7 and FRS12 function as transcriptional repressors and define a distinct regulatory branch involved in growth and flowering control (Figure 3) [17,73]. These two proteins form a repressor complex and the complex could negatively regulate flowering time by suppressing the expression of *GIGANTEA (GI)* and *PIF4* (Figure 3, Table 1) [17]. Consistent with this role, the *frs7 frs12* double mutant flowers earlier than wild-type plants under short day conditions and also displays elongated hypocotyls, reflecting de-repression of pathways controlling photoperiodic growth and developmental timing [17]. Functional analyses further indicate that FRS7 plays the dominant role, whereas FRS12 acts as a partially redundant paralog (Figure 3) [17]. Mechanistically, the FRS7/FRS12 complex recruits the co-repressor NINJA to mediate transcriptional silencing (Figure 3) [73].

### 4.3. Divergent Members Associated with Signaling Crosstalk: FRS3, FRS5 and FRS9

FRS3, FRS5 and FRS9 represent a more divergent set of family members whose available evidence mainly comes from molecular interaction data rather than direct functional validation (Figure 3) [25,26]. FRS3 was identified in protein interaction networks through its association with JAZ3, a repressor in Jasmonate signaling, and with ZML2, a GATA family transcription factor (Figure 3) [25,74]. These proteins might participate in crosstalk between light signaling and jasmonate-related regulatory pathways, although direct genetic evidence is still lacking (Figure 3) [74]. FRS9 is particularly unusual within the family and has been proposed to act through a non-canonical mechanism (Figure 3) [25,26]. The dissimilarity in structure indicates that these proteins may rely more on protein–protein interactions than on direct promoter binding, thereby differing from canonical FRS activators. Thus, FRS3, FRS5 and FRS9 may represent an interaction-centered branch of the FRS family, but their downstream targets and physiological relevance remain to be defined.

### 4.4. Activator-like Members with Limited Functional Validation: FRS6, FRS8, FRS10, and FRS11

FRS6 and FRS8, which belong to Subgroup II, possess the complete domain organization typical of full-length FRS proteins and are therefore generally regarded as putative transcriptional activators (Figure 3) [25,26]. Both *frs6* and *frs8* T-DNA insertion mutants flower early, suggesting that FRS6 and FRS8 contribute to photoperiod-mediated repression of flowering [25]. Despite their structural similarity to FHY3 and FAR1, direct evidence for their target genes and underlying molecular mechanism is still not clear. FRS10 and FRS11 are members of Subgroup V (Figure 3) [25,26,57]. At present, little is known about their molecular interactions, transcriptional targets, or loss-of-function phenotypes (Figure 3) [25,26]. Their persistence in the genome nonetheless suggests that they may perform specialized or context-dependent roles that have not yet been captured by standard developmental assays. Together, these members highlight that structural conservation does not necessarily imply functional conservation, and that direct target identification and mutant analyses are needed to define their roles.

### 4.5. Truncated Modulators in the FRF Subgroup

The FRF Subgroup includes FRF1 to FRF4. These proteins are truncated homologs that keep only part of the FAR1-related region and do not contain the transposase or SWIM domains found in full-length FRS proteins (Figure 3) [26,27]. Because of this reduced architecture, FRF proteins are not thought to function as conventional transcriptional activators [27]. Instead, they have been proposed to modulate FHY3/FAR1-dependent transcription by competing for binding to FBS-containing promoters without supporting transcriptional activation (Figure 3) [26]. Consistent with this model, elevated FRF levels would be predicted to attenuate FHY3/FAR1-mediated transcription and therefore resemble a dominant negative mode of action [27]. Rather than representing non-functional remnants, these truncated proteins appear to add an additional regulatory layer to the family by buffering or restricting the activity of full-length members [26]. Thus, the FRF Subgroup illustrates how domain loss may generate modulatory functions and contribute to developmental-stage or tissue-dependent control of FHY3/FAR1-mediated gene expression.

## 5. Conservation and Diversification of the *FHY3/FAR1* Gene Family Across Plant Species

### 5.1. Monocotyledonous Species

Monocotyledonous species provide important evidence for the conservation and diversification of *FHY3*/*FAR1* gene family members in cereal crops. In maize, around 15 *ZmFARL* genes have been identified and annotated (Table 2) [29,75]. Expression analyses indicate that some members may participate in inflorescence and kernel development [29,75]. A later genome-level survey reported 24 *FHY3*/*FAR1* members in maize, a discrepancy that likely reflects differences in screening criteria and counting strategies, including the use of alternative splice isoforms and broader inclusion of borderline members (Table 2) [76]. Comparative genome-level data also indicate substantial variation in *FHY3*/*FAR1* gene copy numbers among grasses, with 12 members reported in rice (*Oryza sativa*), 24 in maize and 14 in sorghum (*Sorghum bicolor*) (Table 2) [76]. These differences suggest that the *FHY3*/*FAR1* gene family has been retained across monocotyledonous genomes but has experienced lineage-specific expansion and contraction, probably reflecting differences in genome duplication history and potential functional divergence.

Functional conservation of the light signaling module is also supported by cross-species complementation evidence [28,75,77,78,79,80,81]. FHY1-like proteins in *O. sativa* can functionally complement the *Arabidopsis fhy1* mutant, indicating that core components of the phyA nuclear import pathway are conserved across divergent angiosperm lineages [39]. In barley (*Hordeum vulgare*), genome-wide analysis of the FRF subfamily identified HvFRF9 as a drought-responsive member, suggesting that truncated FAR1-related regulators may also contribute to stress adaptation in monocotyledonous crops (Table 2) [37]. In *Dendrobium* species, comparative genomic analyses revealed a marked expansion of orchid-specific *FAR1*/*FRS* lineages, especially Orchid-clade-1, with *Dendrobium* genomes containing an average of 51 homologs and *D. catenatum* carrying as many as 155 members (Table 2) [82]. This expansion was proposed to facilitate adaptation to shaded epiphytic habitats, where canopy-filtered light is enriched in far-red wavelengths and PHYA-mediated light signaling becomes particularly important [82]. Together, these findings indicate that monocotyledonous *FHY3*/*FAR1* and *FRF* members have retained ancestral light-associated regulatory functions while acquiring species-specific roles in development and abiotic stress responses (Table 2).

### 5.2. Dicotyledonous Species

Dicotyledonous species show extensive expansion and functional diversification of the *FHY3*/*FAR1* gene family. In soybean (*Glycine max*), 49 *GmFRS* genes have been identified and classified into seven phylogenetic subgroups (Table 2) [28]. In quinoa (*Chenopodium quinoa*), an allotetraploid species, the family has expanded to 87 *CqFAR1* genes, consistent with the contribution of genome duplication to gene family expansion (Table 2) [30]. In ginseng (*Panax ginseng*), 59 *PgFAR1* genes have been identified, several of which respond to methyl jasmonate treatment, suggesting possible involvement in hormone-responsive regulation and secondary metabolism (Table 2) [31]. In *B. napus*, however, only 21 *BnFAR1* genes have been identified, a number comparable to that in *Arabidopsis*, despite its allotetraploid genome, suggesting limited expansion or possible gene loss after polyploidization (Table 2) [83]. Cross-species comparisons therefore indicate that polyploid or duplication-rich dicotyledonous species often contain expanded FHY3/FAR1 repertoires, although the biological functions of most duplicated members remain to be experimentally validated.

In the tea plant (*Camellia sinensis*), 25 *CsFHY3*/*FAR1* genes were identified and classified into five phylogenetic subgroups, with conserved motif and domain analyses supporting preservation of core FHY3/FAR1 structural features (Table 2) [77]. Most *CsFHY3*/*FAR1* genes are downregulated under cold stress at 4 °C, whereas several members respond to methyl jasmonate or shade treatment, suggesting roles in light adaptation, hormone signaling and stress responses (Table 2) [77]. In poplar (*Populus trichocarpa*), 51 *FHY3*/*FAR1* gene family members have been identified and classified into four phylogenetic subgroups, with duplication analyses suggesting contributions from whole-genome and local duplication events (Table 2) [84].

Several dicotyledonous studies have also begun to connect *FHY3*/*FAR1* genes with specific physiological or metabolic processes. In *Eleutherococcus senticosus*, four *FHY3*/*FAR1* family members were predicted to bind the promoter of *FPS*, which encodes farnesyl diphosphate synthase, a key enzyme involved in triterpenoid saponin biosynthesis, and these genes respond to changes in light quality (Table 2) [85]. This finding suggests a possible link between *FHY3*/*FAR1*-mediated light signaling and specialized metabolite biosynthesis. In tomato (*Solanum lycopersicum*), SlFHY3 cooperates with SlHY5 to enhance cold tolerance through integration of myo-inositol and light signaling (Table 2) [86]. In *Eucalyptus grandis*, 33 members of the *FHY3*/*FAR1* gene family were identified, many of which respond to salt and temperature stress, with salt treatment inducing the expression of several members in leaves and low temperature generally associated with reduced expression (Table 2) [32]. Genome-wide studies in walnut, potato, grape and peanut have further confirmed that *FHY3*/*FAR1* members are widely distributed across dicotyledonous species and show lineage-specific variation in gene number, expression patterns, and potential biological function [78,79,80,81].

Cross-species comparison shows different evolutionary patterns in monocot and dicot lineages. Dicot genomes generally show greater variation in copy number and stronger lineage-specific amplification. In contrast, the monocot repertoires reported so far are relatively small, except in *D. catenatum* (Table 2) [80,82]. This pattern suggests that dicot lineages, especially those with recent polyploid histories, are more likely to retain duplicated *FHY3*/*FAR1* copies. Dicots tend to show more variable and recent expansion patterns, whereas monocots more often show signatures of ancient duplication, constrained retention, and lineage-specific rearrangement or loss.

The relatively large expansion is likely governed not only by the occurrence of whole-genome duplication (WGD), but also by post-polyploid retention and fractionation. Both *A. hypogaea* and *C. quinoa* achieved expansion through polyploidization, and *C. quinoa* exhibits segmental duplication (Table 2) [30,78]. Polyploidy alone does not ensure this large expansion. The limited retention in *B. napus* supports this point [83]. In *D. catenatum*, the *FHY3*/*FAR1* family lacks evidence of recent polyploidy yet has proliferated extensively, likely through segmental rearrangements and selection under shaded epiphytic habitats [82]. Together, these cases suggest that duplication, chromosomal dynamics, ecological pressure, and differences in duplicate retention may jointly shape the size of the *FHY3*/*FAR1* gene family.

### 5.3. Early Land Plants and Broader Evolutionary Implications

The *FHY3*/*FAR1*-related family is not restricted to flowering plants. Comparative transcription factor analyses identified FAR1 as a land plant-associated family present across early and later plant lineages [33,36,87]. Consistent with this distribution, comparative analyses of red/far-red light signaling components inferred that FHY3/FAR1-related components are present in bryophytes, suggesting that FHY3/FAR1-related regulators are consistent with a possible ancient origin [33]. Broader reannotation of *FAR1* genes further supports the view that this MULE-derived regulatory family was retained and structurally diversified in multiple plant lineages [36].

Overall, studies across monocotyledonous species, dicotyledonous species and early land plants indicate that the *FHY3*/*FAR1* gene family combines conservation in angiosperms with possible deeper ancestry and extensive lineage-specific diversification [29,31,76,85,88]. Conserved domain organization and phylogenetic clustering suggest preservation of an ancestral transcriptional regulatory framework, whereas differences in copy number, chromosomal distribution, duplication history and expression profiles imply repeated functional specialization after gene duplication [10,29,31,77,78,79,80,81,84,88,89,90]. Current evidence supports conserved roles in light signaling, together with expanded functions in plant architecture [15], chloroplast development [12,54], abiotic stress tolerance [23], hormone responses [52,58,64] and specialized metabolism (Table 2) [12,20]. Future studies should move beyond genome-wide identification and expression profiling toward direct functional validation of individual *FRS* and *FRF* members, especially in crops, woody plants and medicinal species.

## 6. Perspective

### 6.1. Integrative Perspectives on FHY3/FAR1 Function

Over the past decades, FAR1 and FHY3 have evolved from being viewed as relatively specialized components of phytochrome A signaling to being recognized as central regulators linking light perception with plant development and environmental adaptation [5,9,25]. As discussed above, *FHY3*/*FAR1*-related proteins participate in chloroplast development, carbon metabolism, flowering, branching, seed dormancy and germination, leaf senescence, immunity, and abiotic stress responses, indicating that they function as transcriptional hubs rather than components of a single linear pathway [18,19,20,22,23,24,53,54]. This integrative role appears to depend on promoter recognition through FBS motifs (Table 1), interaction with pathway-specific transcriptional partners, and signal-dependent regulation of protein abundance or post-translational state [18,19,20,22,23,24,53,54]. These mechanisms provide a flexible framework through which FAR1 and FHY3-related proteins integrate light, hormonal, developmental, and stress signals into context-specific transcriptional outputs.

The evolutionary origin of this family shows one of its most distinctive features. FHY3/FAR1 proteins were derived from MULE transposases and represent a well-documented case of transposable element exaptation in plants (Figure 1a) [2,9,38,91]. During molecular domestication, transposase-derived modules were repurposed for host gene regulation: catalytic activity required for transposition was lost, whereas sequence-specific DNA-binding function may have been retained or remodeled for the recognition of host cis-regulatory elements. In FHY3/FAR1 proteins, this activity is mediated by the N-terminal FAR1 DNA-binding domain, which recognizes FHY3/FAR1-binding sites in target promoters (Figure 1a) [9,26,34]. This evolutionary transition highlights how plant genomes can convert mobile elements into stable regulatory components.

The molecular mechanism of FHY3/FAR1 action may be further considered in terms of recent transcription factor models. Current evidence supports a classical framework in which the N-terminal FAR1 domain mediates DNA binding, the central region contributes to dimerization or protein interactions, and the C-terminal SWIM-containing region is required for transcriptional activation [1,26,34,92]. However, recent conceptual advances suggest that transcriptional specificity often emerges from the combined action of structured DNA-binding domains and flexible or intrinsically disordered regions (IDRs) that mediate transient, multivalent interactions with partner factors and the transcriptional machinery [92]. Whether this principle applies directly to FHY3/FAR1 remains unknown. Applied cautiously to FHY3/FAR1, this model suggests that promoter recognition by the FAR1 DNA-binding domain may provide initial chromatin anchoring, whereas putative IDRs together with other central or C-terminal regions may help shape the final regulatory output through context-dependent protein interactions. Consistent with this possibility, UniProt annotations suggest several FHY3/FAR1 proteins contain predicted intrinsically disordered regions [93]. The broad interaction network of FHY3/FAR1 proteins is also consistent with a possible role in the local assembly of chromatin-associated regulatory complexes [21]. In the ‘emergent specificity’ model, neither the DNA-binding domain nor the IDRs alone would be sufficient to reproduce the chromatin localization and regulatory activity of the full-length proteins [92]. Instead, the full regulatory output may arise from the combined effects of DBD–DNA contacts and multivalent interactions mediated by IDRs and other central or C-terminal regions [92]. The view may also help explain the functional divergence of truncated family members, including repressors or competitive modulators [15,21,25,26,94]. However, these observations are still indirect. Whether FHY3/FAR1 proteins contain *bona fide* functional IDRs, and how such regions contribute to transcriptional regulation, will need to be tested directly.

The evolutionary transition may reflect modular refinement during the evolution of the *FHY3*/*FAR1* gene family. In this process, the ancestral transposase catalytic core might have been lost or degenerated, whereas DNA binding and protein interaction modules are retained. This interpretation is consistent with their extensive partner networks, but direct evidence linking specific domain configurations to integrative capacity is still limited.

Within this modular framework, FHY3/FAR1 proteins appear to act as system integrators. They connect light signaling with the circadian clock, hormonal pathways, developmental timing, and stress adaptation through interactions with regulators such as HY5, PIFs, EIN3, SPLs, and TOC1 (Figure 2) [16,18,19,21,22,94].

### 6.2. Knowledge Gaps and Future Directions

Although much has been learned, we still know relatively little about the functions of the family as a whole. Several *Arabidopsis* members, including FRS6, FRS8, FRS9, FRS10, and FRS11, remain poorly studied, and the FRF family still needs stronger genetic and biochemical evidence [25,26]. In addition, post-translational regulation of FHY3/FAR1 remains insufficiently resolved. Available evidence suggests that the protein abundance and activity of FHY3/FAR1 are dynamic, but the roles of phosphorylation, ubiquitination, and proteasome-associated turnover of them are still unclear [26,44]. In particular, the mechanisms underlying the decline of FHY3/FAR1 protein abundance during aging or under shade conditions are poorly understood, despite their likely importance for senescence-associated regulation and plant architecture [16,18].

In plants, research has expanded rapidly but has remained largely descriptive. Genome-wide surveys have identified *FHY3*/*FAR1* gene family members in tea, peanut, eucalyptus, walnut, potato, maize, quinoa, ginseng, grape, barley, soybean and other taxa [28,29,30,31,32,37,77,78,79,80,81]. These studies collectively indicate that the family is broadly conserved and often expanded [28,84], but functional validation through reverse genetics, genome editing, and physiological analysis is still limited.

The expansion dynamics of the *FHY3*/*FAR1* gene family is still mostly inferred rather than experimentally tested. It remains unclear whether duplicated members in rapidly expanding lineages have gained new functions, partitioned ancestral functions, or provided dosage buffering. Future studies should combine phylogenomic analysis with reverse-genetic validation in species with contrasting expansion histories, such as *A. hypogaea* and *D. catenatum*. Such work will help determine whether convergent expansion reflects similar selective pressures or shared genomic tendencies.

Future work should therefore move beyond gene inventories toward dynamic regulatory analysis. Chromatin-based methods such as ChIP-seq, and DAP-seq will be essential for defining direct targets, while transcriptomics, proteomics, and epigenomic profiling under defined environmental conditions will help distinguish primary from downstream responses. Because *FHY3*/*FAR1* gene functions are likely to be tissue- and stage-dependent, single-cell and spatial proteomics should provide important resolution beyond bulk transcriptomics [95]. Structural and biophysical studies will also be needed to test whether the emergent specificity framework is mechanistically applicable to FHY3/FAR1 proteins [92].

Functional redundancy is likely to be a central obstacle. Many plant genomes contain multiple FRS/FRF homologs, and single mutants may show weak or no phenotypes because of compensation by closely related members. Multiplex CRISPR/Cas9 approaches will therefore be important for dissecting redundant subclades, whereas cis-regulatory editing may provide a more practical way to fine-tune FHY3/FAR1 activity without the pleiotropic costs often associated with constitutive overexpression or complete knockout [26,96]. This is particularly relevant for translational applications, because *FHY3*/*FAR1* orthologs have already been associated with shade avoidance, branching, flowering time, cold tolerance, salt responses, drought responses, and specialized metabolism in several species [32,37,85,86]. The modular, transposase-derived nature of this family may also offer opportunities for synthetic transcription factor design in plants [97].

Overall, the next stage of *FHY3*/*FAR1* gene family research will depend on integrating evolutionary analysis, mechanistic studies, functional genomics, genome editing, and plant physiology. Future work should clarify how different family members acquire specific functions, how they work together within regulatory networks, and how their activities could be used to improve plants under changing environmental conditions [2,26,29,75,76,79].

## 7. Conclusions

FHY3/FAR1 proteins illustrate how transposase-derived factors can be domesticated into plant transcriptional regulators. Current evidence shows that this family is broadly conserved across angiosperms and contributes to light signaling, development, stress responses, hormone pathways and specialized metabolism.

Future research should prioritize direct functional validation, the identification of regulatory targets and mechanistic analysis of family-specific transcriptional control. Such work will clarify how *FHY3*/*FAR1* gene family members achieve regulatory specificity and may support their use in plant improvement and metabolic engineering.

## Figures and Tables

**Table 1:** The direct targets and regulatory modes of the FHY3/FAR1 protein family. The table summarizes representative target genes, biological processes, regulatory proteins, modes of regulation and cis-elements, highlighting how FHY3/FAR1 and related FRS members control light signaling, hormone responses, flowering, branching and senescence.

Target Gene(s)	Biological Process	Regulated by	Regulation Type	Cis-Element
*FHY1*, *FHL*	PhyA nuclear import	FHY3/FAR1	Activation	FBS
*CCA1*	Circadian clock	FHY3/FAR1	Activation	FBS
*HEMB1*	Chlorophyll biosynthesis	FHY3/FAR1	Activation	FBS
*ARC5*	Chloroplast division	FHY3/FRS4	Activation	FBS and FBL
*ABI5*	ABA signaling	FHY3/FAR1	Activation	FBS
*SMXL6*, *SMXL7*	SL signaling	FHY3/FAR1	Activation	FBS
*GI*, *PIF4*, *PIL1*	Flowering/clock output	FRS7/FRS12	Repression	FRB1, FRB2, FRB3
*WRKY28*	SA biosynthesis	FHY3/FAR1	Repression	FBS
*CLV3*	Meristem stem cell maintenance	FHY3	Repression	FBS
*SEP2*	Floral organ identity	FHY3	Activation	FBS
*SPT*	Seed dormancy/germination	FHY3	Activation	FBS
*RVE2*, *RVE7*, *phyB*	Seed dormancy/germination	FHY3	Repression	FBS
*ACO1*	Ethylene biosynthesis	FHY3	Activation	FBS
*ISA2*	Starch metabolism	FHY3/FAR1	Activation	FBS
*COP1*	UV-B signaling	FHY3	Activation	FBS
*PHR1*	Phosphate starvation response	FHY3/FAR1/EIN3	Activation	FBS
*MIPS1*	Myo-inositol biosynthesis/ROS	FHY3/FAR1	Activation	FBS

**Table 2:** *FHY3*/*FAR1* gene family members in representative plant species. The table summarizes the gene numbers, predicted roles, validated functions and subgroup classification of *FHY3*/*FAR1* family members across plant species, highlighting their conserved roles in light signaling, development and stress responses. The data shown are based on the classification schemes used in the corresponding original studies. An asterisk (*) indicates an experimentally validated function. Unmarked entries indicate functions predicted from expression profiling or transcriptomic analyses. (q) denotes functions inferred from phylogenetic clustering, sequence similarity, or functional annotation without experimental validation.

Species	Gene Number	Subgroups (Clades)	Putative Functions
*A. hypogaea*	246	3	Growth regulation (Pod development); stress responses (q)
*A. thaliana*	18	6	Light signaling *; Photomorphogenesis *, chlorophyll biosynthesis *, ABA signaling *, flowering regulation *, ROS homeostasis *, leaf senescence *, plant immunity *
*B. napus*	21	4	Shading response; low-temperature response; silique development; hormone signaling
*C. quinoa*	87	5	Seed germination (q); spike sprouting; growth and development; light signaling; hormone response (ABA, GA, MeJA); abiotic stress response
*C. sativus*	20	3	Photomorphogenesis; Flowering; abiotic stress; hormone responses
*C. sinensis*	25	5	Biotic/abiotic stress; light signaling (q); hormone response (MeJA) (q); shading response; photomorphogenesis;
*D. catenatum*	155	7	Light signaling
*E. coracana*	42	5	Light signaling salt stress response
*E. grandis*	33	3	Salt and temperature stress *; light signaling (q)
*E. senticosus*	21	5	Light quality response; secondary metabolism (predicted saponin synthesis regulation); growth and development (q)
*G. max*	49	7	Light signaling; shade avoidance response; stress adaptation (q); developmental regulation; hormone response (ABA, JA)
*H. vulgare*	30	3	Light signal transduction (q); growth and development (q); stress responses (q);
*J. sigillata*	61	5	Growth and development; hormone signaling; stress responses
*O. brachyantha*	32	3	Light signaling (q); Photomorphogenesis (q); stress tolerance (q)
*P. ginseng* C. A. Mey.	59	6	Secondary metabolism (ginsenoside); hormone signaling (MeJA); growth and development
*P. trichocarpa* Torr. & Gray	51	4	Light response; growth and development; hormone and stress response
*P. vulgaris*	27	3	Salt and drought stress; light signaling; abiotic stress response
*S. tuberosum*	20	6	Growth and development *; abiotic stress responses; light signaling
*V. vinifera*	43	7	Salt stress resistance; JA response; light signaling; growth and development
*Z. mays* ‘B73’	15/24	5	Light signal transduction; growth and development; stress responses;

## Data Availability

No new data were created or analyzed in this study.
